# Do public health expenditures affect maternal and child health in Madagascar?

**DOI:** 10.1186/s13561-023-00462-7

**Published:** 2023-10-18

**Authors:** Marilys Victoire Razakamanana, Voahirana Tantely Andrianatoandro, Tiarinisaina Olivier Ramiandrisoa

**Affiliations:** 1 Centre de Recherche pour le Développement (CRD), Université Catholique de Madagascar Ambatoroka, Catholic University of Madagascar, Antananarivo 101, Ambatoroka, BP 6059 Madagascar; 2 Unité Mixte de recherche Internationale “Soutenabilité et Résilience” (UMI SOURCE), IRD- Université Paris-Saclay, Paris, France; 3 Centre d’Etudes Economiques (CEE), Université d’Antananarivo, Ambohitsaina , Antananarivo, Madagascar

**Keywords:** Madagascar, Public health expenditure effects, Maternal and child health care, Panel data

## Abstract

**Background:**

Previous studies have argued that the relationship between health expenditures and health outcomes is more significant among the poor than the non-poor. However, public spending alone does not improve health status. Quality of governance is considered not only as an important determinant of health outcomes but also of the efficiency of public expenditure on health. In low-income countries, barriers to quality service provision can be observed, which may explain the effects of health expenditures. Therefore, this paper aims to identify the relationship between health expenditures and maternal and child mortality in Madagascar and the potential bottlenecks in the flow of funds for maternal and child health.

**Methods:**

Using panel data, fixed and random effect models for the 22 regions of Madagascar over the period 2010 to 2017 were used. Then bottlenecks related to the flow of funds for maternal and child health were identified.

**Results:**

The results reveal that, on the one hand, funding for maternal health, mainly constituted by equipment endowments for health facilities, significantly contributes to the improvement of maternal health (-0.35; p-value = 0.00). On the other hand, child health financing, often realized through transfers of funds to the health system, does not affect children’s health (0.22; p-value = 0.88). The bottleneck analysis illustrates that the transferred funds can suffer from delay or misappropriation and only few parts reach beneficiaries.

**Conclusions:**

Equipment endowments contributed more to health improvement and would be more effective than monetary financing.

## Introduction

### Background

Public funding, particularly in low-income countries, has an important role in achieving universal health coverage (UHC) and improving health. Indeed, investments in health and infrastructure contribute improving health status [1–4]. The effects are much greater in low-income countries than in high-income countries, and the return on health investment is also higher for low-income countries [5, 6]. Examining the case of low-income countries, Gupta et al. (2002) and Rana et al. (2018) found that there is a significant negative relationship between health expenditures and child mortality [5, 6]. However, some studies have shown that there are no significant effects on child mortality [7, 8], on maternal mortality [9] or on either child or maternal mortality [10, 11]. Filmer and Pritchett (1999) have argued that this relationship depends on the composition and efficiency of health expenditures [8]. Indeed, health expenditures can improve health status by providing effective health services and quality of governance is also essential [8, 12].

Governance is a key to improving health sector performance and achieving UHC [13]. Measures of governance can be rule-based determinants such as the presence of standards and laws and / or outcome-based performance measures such as health worker absenteeism, proportion of government funds reaching district facilities, misuse of resources, proportion of insufficient funds [13]. In low-income countries, the values of these indicators may indicate insufficient good governance, partly explaining the effects of health expenditures [14].

Madagascar is a low-income country where access to health care remains difficult [15]. In 2019, according to the Human Development Index of the United Nations Development Programme (UNDP), the country was ranked 164th out of 189 countries and according to the World Bank data, over 75% of the population earns less than US$1.90 per day (in purchasing power parity). Between 2015 and 2019, the budget needed to ensure UHC was US$1,892 million per year and 56.3% of this fund should have been allocated to maternal and child health programmes [16]. However, the funding actually allocated to health remains low. It is below the threshold of the humanitarian emergency which represents the minimum threshold to ensure the basic functioning of the health system. Indeed, in 2015, the total health funding was US$510 million, while the minimum required for basic functioning was estimated at US$733 million [16]. Thus, funds available for health constituted only 70% of this minimum.

In Madagascar, according to the World Health Organization (WHO), in 2019, 10% of the health expenditures come from the private sector, 41% from households, and 48% from the government. Public health expenditures are mostly financed by external funds. They were between 58% and 72% from 2009 to 2015. The budget allocated for health is below 10% of the state budget.

The major health problems in Madagascar remain the resurgence of epidemic diseases such as plague and polio, high maternal, neonatal and child mortality, high level of malnutrition, and increase of non-communicable diseases [17]. Maternal mortality rate remains high; it varied from 488 per 100,000 live births in 1997 [18] to 384 per 100,000 live births in 2019. At the regional level, deaths range from 402 to 594 per 100,000 live births [19]. Child under-five mortality rate has improved since 2000: from 109 deaths per 1,000 live births in 2000 to 50.6 deaths per 1,000 live births in 2019 (data of WHO). However, this rate is still above the global average (37.7 per 1,000 live births in 2019). Moreover, at the regional level, very wide disparities appear. The highest level of neonatal mortality in 2017 is estimated at 327‰ and the lowest is 2‰ [20].

This paper aims to assess the effects of health expenditures on maternal and child health considering the case of Madagascar. Is there a relationship between health expenditures and maternal and child mortality? Considering the regional disparities in mortality, what are the potential bottlenecks in the flow of funds for maternal and child health? Indeed, some studies showed that in maternal and child health programmes, these are the bottlenecks of concern: administrative process, funding flow, access, and clinical practices [21–24]. In our case, the funds allocated to the regions should depend on their characteristics. Indeed, some regions are much more exposed to diseases than others, such as the eastern regions of Madagascar. Then, the absence or presence of these bottlenecks can explain the effects of health expenditures.

In Madagascar, no study on this subject has yet been carried out. In 2019, the objective was to have a maternal mortality rate of 300 per 100,000 live births and a child mortality rate equal to 35 per 1,000 live births [19]. However, even by 2021, these targets had not been met. This situation could be linked to insufficient health funding and its management. Based on the effects of health spending on maternal and child health, the results will be used to advocate the prioritization of Madagascar’s health sector and the optimal allocation of funds to achieve these objectives.

## Methods

Madagascar is administratively subdivided into 22 regions and 119 districts. Then, different levels constitute the health system in Madagascar: the central level, the regional level, and the district and community levels. The Ministry of Public Health (MOH) is the central coordinator. At the regional level, the MOH is represented by the Regional Health Offices (RHO) and the District Health Offices (DHO). They implement and supervise the health programmes.

Graphical and empirical analysis are performed to study the relationship between health expenditures and maternal and child mortality. Bottlenecks analysis is focused on funds for child health immunization and grants for maternal health.

### Analysis of the effects of health expenditures on maternal and child health at the national level

National health expenditures and mortality data from 2000 to 2017, are taken from the WHO database. Thus, because of the low number of observations, a graphical analysis is carried out. The figure shows the evolution of healthcare expenditures (per capita USD) and the maternal (per 100,000 live births) and child mortality rate (per 1,000 live births) over time (2000–2017). Health expenditures are classified according to their origin: by public and private funds. Public financing is composed by government funds for health and external financing distributed by the government while private funding is the household contribution and voluntary prepayments.

### Analysis of the effects of health expenditures on maternal and child health at the regional level

As the distribution of funding differs from one region to another, it is important to verify whether regions that have spent more on health have a better health indicators than those with a lower level of expenditures.

Health expenditures and health indicators of the 22 regions are available over the period 2010‒2017. Then, the effects are determined from fixed and random effect models, and are presented as follow:

$${Y_{it}}\, = \,f\left( {{H_{it}},\,\,{X_{it}}} \right).$$

Where,

*Y*_*it*_ is the variable representing the maternal and child mortality for region *i* at period *t*. Health indicators at the regional level come from the MOH database (Health Statistical Yearbooks, available from 2010 and whose last available report was published in 2018).

*H*_*it*_ represents the health expenditures per capita of region *i* at period *t*. Regional health expenditures per capita come from the Ministry of Finance and Budget’s Integrated Computerized Public Finance Management System (SIG-FP) from 2010 to 2017. It includes public expenditures and external funds that flow through public channels into national health systems. Promotive or preventive spending for maternal and child health from Technical and Financial Partners (TFPs) funds is included in these data, such as for the purchase of mosquito nets, vaccines, delivery kits, etc. It is worth nothing that there is no separate information about Maternal Health Expenditures and Child Health Expenditures. They are included in the *H*. This is a limitation of our study, as the data in Madagascar do not allow to consider funds for maternal and child health separately. However, there are cross-cuttting activities that could affect both maternal and child health (e.g. the fight against malaria). With private and households expenditures not being included, the aim is to see whether the allocation of public and external funds effectively improves the health of the mother and child.

*X*_*it*_ are the control variables. Similar to Novignon et al. (2012), variables related to disease prevention and disease prevalence are considered as control variables [2]. The maternal health indicators include the family planning utilization *F*_*it*_, the prenatal consultation *PC*_*it*_, and the delivery at health facilities *D*_*it*_. Child health indicators are the prevalence of the three main childhood diseases (malaria *M*_*it*_, acute respiratory infections including pneumonia *ARI*_*it*_, and diarrhea *Di*_*it*_) and the immunization coverage. Family planning utilization F_it_, the prenatal consultation PC_it_, and the delivery at health facilities D_it_ can positively affect maternal health, then decrease maternal mortality [2]. Immunization coverage can also reduce child mortality. However, malaria, ARI and diarrhoea are the main causes of child mortality in Madagascar. Then, as social variables, the gross enrolment rate *E*_*it*_ by region is introduced. Indeed, according to Akinkugbe and Mohanoe (2009), enrolment rates can affect health status [1]. This variable is from the Madagascar National Statistics Institute database (2018). Finally, the International Wealth Index (IWI) W_it_ is considered. This variable comes from the Global Data Lab. The IWI is based on twelve assets. These assets include seven consumer durables (possession of a TV set, a fridge, a phone, a bike, a car, a cheap utensil and an expensive utensil), access to two public services (water and electricity) and three housing characteristics (number of sleeping rooms, quality of floor material and of toilet facility). The IWI is scored from 0-100, with 0 representing households with none of the assets and 100 representing households having all assets.

Since the regions observed belong to the same country, we assume that the coefficients of the variables are similar. Thus, the only sources of heterogeneity can come from the constant. Therefore, two cases can be distinguished: the case which individual effects are deterministic constants terms (fixed-effects model) or the case which they are assumed to be the realizations of random variables of finite expectation and variance (random effects model). The Hausman specification test confirm whether it is a fixed or a random effect model. Then, the models are as follows:

$${Y_{1it}} = {\alpha _{1it}} + {\beta _1}Ln{H_{it}} + {\beta _2}{F_{it}} + {\beta _3}P{C_{it}} + {\beta _4}{D_{it}} + {\beta _5}{E_{it}} + {\beta _6}{W_{it}} + {\varepsilon _{1it}}$$

$${Y_{2it}} = {\alpha _{2it}} + {\delta _1}Ln{H_{2it}} + {\delta _2}{M_{it}} + {\delta _3}AR{I_{it}} + {\delta _4}D{i_{it}} + {\delta _5}{E_{it}} + {\delta _6}{W_{it}} + {\varepsilon _{2it}}$$

*Y*_*1it*_ for maternal health and *Y*_*2it*_ for child health; *α* is the individual effect; β and δ are the coefficients and *ε* the error term.

To test the robustness of our results, Generalized Moments Method (GMM) is used. The advantage of this method is that it solves the problems of simultaneity bias, reverses causality and omitted variables [25]. The GMM is used to correct the endogeneity in the weak sense but not in the strong sense. Indeed, variables cannot be affected by future shocks. It was developed by Holtz-Eakin, Newey and Rosen (1988) and Arellano and Bond (1991) [26, 27]. In this paper, system GMM is used. The estimation of the system GMM combines the first difference equations with the level equations where the variables are instrumented by their lagged values.

### Bottlenecks analysis related to the flow of immunization fund and maternal health financing

The bottlenecks analysis concerns:

- The transfer delays: A delay occurs if the difference between the expected transfer date and the date of the funds reception exceeds ten days knowing that the activities schedule iscommunicated in advance by the MOH.
- The funding gap and reallocations: A funding gap is considered when the amounts obtained by the beneficiaries are less than the budget. In this case, the expenditure is recorded in the central level account as committed, although it has not reached the beneficiaries. Reallocations occur when beneficiary reallocates the funds they received to solve other problems.
- The lack of monitoring or control of funds: Monitoring must be carried out systematically. According to WHO, health facilities, DHO and RHO should respectively be monitored every two, three and six months.

The bottleneck analysis is based on two types of data:

- Survey data on the use of funds for immunization. These data are from UNICEF (2015) and from the results of the GAVI Financing Audit (GAVI, 2017). These data are the only ones available but they are representative. In fact, the UNICEF surveys covered ten regions of Madagascar. Two districts per region were selected, one with a high immunization performance and one with a low performance. Similarly, at the district level, four health facilities were selected, two had high immunization performance and two were among the lowest. UNICEF survey and GAVI financing audit consider all levels of the health system.

Information on the dates on which funds were sent and received is available in these data from UNICEF (2015). To identify delays in receiving funds, we calculated the difference between these two dates. The UNICEF (2015) survey also asked whether any reallocations had been made. Then, data on the number of checks carried out at health facilities, the DHO and the RHO are also available. A comparison between this information and the WHO standard was then made. For the funding gap, information in the GAVI audit report carried out in 2017 were used.

- Interviews on the use of government funds for maternal health care that were conducted in 2019 with some officials at the RHO and the DHO. In fact, the 2019 Supplementary Finance Act allocates a budget line for “maternal and child survival” including safe motherhood, adolescent reproductive, and family planning services. Two regions where maternal mortality remains high (Vatovavy Fitovinany and Atsimo Atsinanana) and two regions among the lowest (Diana and Analamanga) were considered.

It was asked whether the managers have ever received in-kind donations or funds for maternal health. How often they receive these funds or donations. When did they last receive funds or donations? What are the obstacles to obtaining them? Questions were asked about the different types of bottlenecks.

## Results

### Graphical analysis

**Fig. 1:**
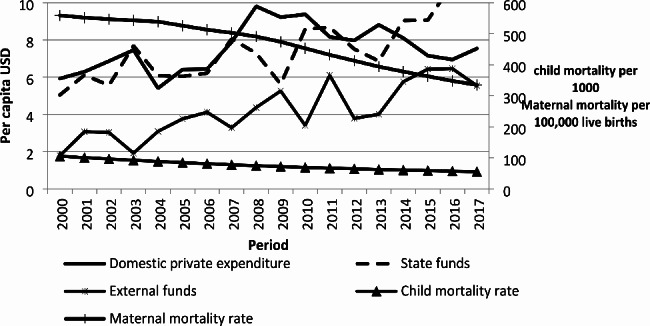
Evolution of health expenditures and its sources of financing from 2000 to 2017. Source: National Health Account, WHO and World Development Indicators, World bank, 2023

The Fig. 1 shows that maternal and child mortality fell between 2000 and 2017. During this period, health expenditures were mainly financed by households (39.7%) and government (39.0%). The external funds decreased in 2002, 2003 and 2010 mainly due to the socio-political crises in Madagascar, and in 2012 due to the transitional government period which isolated the country from the international communities. External funds are used to finance some specific programmes such as Mother and Child Health Week (MCHW), Routine Immunization Intensification Day or programme for the management of childhood diseases at the community level. Government funds varied slightly between 2000 and 2017. They are mainly intended for the health employee remuneration and for the current operations such as the purchase of office consumables [29].

### Relationship between health expenditures per capita and maternal and child mortality at the regional level

The results in Table 1 firstly show that health expenditures are unequally distributed across the 22 regions. As indicated, the health expenditures in the following table are composed of public expenditures and external funds that flow through public channels into national health systems. Maternal and child health have been the priorities of the Malagasy government since 2000. However, the funds are distributed according to the characteristics of the regions. Thus, Analamanga, including the capital and where the central level of the Ministry of Health is located, is among the regions with the highest health expenditures. Then, it should also be noted that there are regions favoured by TFPs. This is, for example, the case with malaria endemic areas in the East of Madagascar (such as Atsinanana), and the mycetoma endemic areas (Boeny) [30]. On the basis of the average annual expenditures per capita from 2010 to 2017, 85.7% of expenditures is allocated to six regions. The other regions receive an insignificant share of this expenditures (between 0.5 and 1.2% of the total). However, health outcomes do not depend on expenditures. Among these six regions, three have high maternal mortality (ranging from 253 to 346 deaths per 100,000 live births) although child mortality in these regions is among the lowest and is less than 10‰. Secondly, among the four regions with the lowest health expenditures (Androy, Sofia, Vakinankaratra, Itasy), the maternal mortality rate is highly variable (between 67 and 248 deaths per 100,000 live births) and the child mortality rate is less than 10‰, except for Sofia (10.71‰). Only five regions (Diana, Analamanga, Itasy, Ihorombe, and Bongolava) have a maternal mortality threshold below 70 per 100,000 live births (SDG3).

**Table 1:** Maternal and child health indicators, average from 2010 to 2017

	Maternal health indicators					Child health indicators				
**Regions**	Health expenditures per capita (USD)	Mortality rate per 100 000 lives births	Contraceptive coverage (%)	Prenatal consultation rate 5%)	Delivery in heath facilities (%)	Child mortality rate (‰)	Immunization coverage (%)	Malaria prevalence (%)	Diarrhea prevalence (%)	ARI prevalence (%)
Boeny	12,333.76	253.55	22	73.68	40.94	6.9	64.35	0.8	2.03	3.77
Atsinanana	11,148.92	336.56	21.16	60.25	23.63	9.5	64.94	1.43	1.07	2.72
Haute Matsiatra	10,767.08	95.94	28.04	51.86	26.87	25.7	65.08	0.11	0.9	1.95
Analamanga	10,320.29	65.38	26.68	37.43	24.89	5.3	58.12	0.05	1.45	4.71
DIANA	10,228.11	24.45	29.17	57.2	28.48	2.5	66.77	0.25	1.55	3.09
Atsimo Andrefana	7,308.65	346.62	13.22	62.12	24.54	9	61.34	0.89	1.31	1.97
Melaky	1,350.69	504.54	12.19	47.86	19.86	11.7	54.72	0.85	0.64	1.18
Betsiboka	981.47	506.81	17.86	62.38	39.39	8.5	54.47	1.16	1.53	2.18
Ihorombe	927.67	56.14	22.13	60.01	20.85	14	64.89	0.81	1.49	2.75
Menabe	782.68	278.57	20.53	59.07	18.46	8.3	64.07	0.69	1.18	1.66
Amoron’Imania	698.93	112.32	23.49	49.82	25.42	31	54.81	0.11	1.03	1.9
Analanjirofo	615.88	350.86	22.38	41.9	18.61	7.2	63.19	0.23	0.42	0.98
Alaotra Mangoro	591.84	139.94	29.73	59.11	28.62	10.5	65.84	0.24	0.94	2.29
Bongolava	587.31	69.28	22.17	63.56	24.66	10.4	66.62	0.25	1.31	2.64
Atsimo Atsinanana	516.74	415.05	10.95	63.44	19.67	16	56.65	2.44	0.92	2.34
Anosy	513.93	412.07	16.39	63.08	23.81	10.3	65.98	0.92	1.32	2.53
SAVA	505.07	133.13	14.88	52.46	29.87	8	61.46	0.39	0.87	1.29
Vatovavy Fitovinany	496.10	572.96	22.98	67.07	22.06	35.3	72.79	1.2	0.92	1.88
Androy	468.67	164.71	8.74	60.47	19.6	7.7	62.08	0.3	1.02	1.67
Sofia	457.45	248.58	14.03	49.83	23.75	10.7	55.02	0.65	0.75	1.13
Vakinankaratra	430.59	92.73	24.15	55.34	24.77	7.4	67.32	0.05	1.07	2.52
Itasy	408.14	67.53	31.24	50.61	33.79	6.6	60.38	0.04	1.07	2.59

Sources : SIG-FP- Ministry of Finance and Budget 2019, Health Statistical Yearbooks, 2010 to 2017, MOH, authors, 2019

The results of our model (Table 2) shows that health expenditures per capita affect maternal mortality but have no significant effect on child mortality. The modalities of financing health activities can explain these results (transfer of funds or endowment of equipment).

Maternal and child mortality and disease prevalence are expected to be low when the preventive care is effective. In our case, the indicators available related to prevention activities at health facilities are on contraceptive coverage, prenatal consultation, delivery in health facilities, and immunization coverage for children. Delivery in health facilities significantly reduce maternal mortality, but contraceptive coverage and prenatal consultation are not significant. The use of these three services remains low and differs by region. Since 2010, the situation has not changed. In fact, the average prenatal consultation rate was from 64.9% in 2010 to 63.3% in 2017 and from 25.9–25.6% for the delivery in the same period. For contraceptive coverage, the average coverage rate is less than 25%. School enrolment also affect maternal mortality: the higher the school enrolment rate, the lower the maternal mortality rate.

As for child health, the average immunization coverage rate is not yet satisfactory. At the regions, it ranges from 54.7–73% over the period 2010‒2017, and at national level, it was 67% in 2010 and 70% in 2017. Therefore, the effect of immunization coverage is not significant. Even though malaria, ARI and diarrhoea are the main causes of child mortality, a rise in malaria prevalence causes a slightly significant increase in mortality, and ARI and diarrhoea do not affect child mortality. It is the same case for school enrolment.

Descriptively, the two regions with the highest IWI values, i.e. the richest, have the lowest maternal and child mortality rates (Analamanga including the capital and DIANA tourist areas of Madagascar, respectively average IWI equal to 40.21 and 29.39). However, Table 2 does not confirm this relationship, the IWI only slightly affects the maternal mortality rate and the relationship with the child mortality rate is not significant.

**Table 2:** Relationship between maternal and child mortality and public health expenditures per capita at regional level from 2010 to 2017 in Madagascar: fixed and random effect model estimates

Indicators	Maternal mortality	Indicators	Child mortality
Health expenditures per capita	-0.35*** (0.00)	Health expenditures per capita	-0.22 (0.88)
Contraceptive coverage	-0.00 (0.54)	Immunization coverage	0.12 (0.42)
Prenatal consultation	0.01 (0.11)	Malaria prevalence	4.87* (0.07)
Delivery rate in health facilities	-0.03** (0.02)	Diarrhea prevalence	-3.61 (0.48)
		ARI prevalence	1.07 (0.37)
School enrolment	-0.01** (0.03)	School enrolment	0.07 (0.36)
International Wealth Index	-0,06* (0,06)	International Wealth Index	-0.17 (0.68)
Constant	5.79*** (0.00)	Constant	-12.72 (0.55)
R2	0.16	R2	0.25
Fisher	3.88*** (0.00)	Fisher	.
Hausman test	17.25*** (0.00) Fixed effect model	Hausman test	6.63 (0.47) Random effect model
Observations	153	Observations	153

Sources: Health Statistical Yearbooks, SIGFP, authors, 2021

Using the GMM method, similar results were obtained. The effects of health spending on maternal mortality are negative and significant (-0.31; p-value = 0.04), while the effects on child mortality are not significant (-21.48; p-value = 0.68). For the two models, the p-value of the Sargan test are higher than the margin of error of 0.05 (respectively 0.87 and 0.55), so, the null hypothesis cannot be rejected, instruments are valid.

### Bottlenecks analysis

#### Bottlenecks related to the flow of funds for immunization

Bottleneck analysis concerns the transfer delay, the use of funds, and the control of the use of the allocated and the received funds. Concerning the transfer delay, the allocated funds generally arrive later than the expected start date of activities. Public funds arrive one to six weeks after the expected date of receipt of funds. They are mainly intended for the purchase of consumables, fuel necessary for the fridge to conserve the immunization products and immunization campaigns. Then, in order to meet the schedule established by the MOH, the heads of health facilities carry out the activities with their own funds.

In general, the received amounts correspond or exceed the forecasted amounts. The budget execution rate for immunization is around 95% at central level and 100% at regional level. There was no reallocation of funds. The received funds are fully spent and considered as insufficient (Authors’ survey, 2019). However, audit carried out by the GAVI in 2017 at central level shows a misappropriation of funds from GAVI Alliance financing between January 2013 and December 2016 amounting to US$769,062, and a misuse of an amount totalling US$866,198 was recorded. This expenditure was recorded centrally and therefore included in our data but was considered ineligible or never reached the beneficiaries.

Controls required by WHO is respected for the RHOs and the DHOs. At the community level, the number of controls led by DHO depends on the distance where the health facilities are located. Consequently, health facilities close to the district are controlled several times a month while health facilities in remote areas have never been monitored [28]. The main cause is insufficient funds to carry out the controls. Insufficient funds due to embezzlement reduce the funds transferred at regional level and, consequently, reduce the effectiveness of activities. These bottlenecks may explain the non-significant effects of the health expenditure on child mortality.

#### Bottlenecks in maternal health grants

As mentioned above, maternal health benefits from very few cash transfers but benefit mainly from material endowments (author interviews, 2019). According to seven DHOs surveyed, these donations are provided by the TFPs (one DHO does not know the source of the received donations).

Donations are not regular but depend on the TFPs. Thus, managers are just informed that they will receive materials in few days. The last donation was in 2018 for one DHO, 2017 for two DHOs and before 2016 for five DHOs. Over the past five years, no monetary funding other than for MCHW has been received. From the RHOs and DHOs surveyed, the received funds consist mainly to finance child health programmes. As said earlier, although the ‘maternal and child survival’ has a specific budget line in the supplementary finance acts; only the central level benefits from this fund. Thus, according to the eight DHOs surveyed, the main bottleneck identified in terms of maternal health is the lack or absence of funds for the maternal health promotion.

## Discussion

Our results are in line with those of Filmer and Pritchett (1999) who studied the case of poor countries including Sub-Saharan African and non-poor countries. According to these authors, the relationship between health expenditures and health outcomes depends on the composition and efficiency of health spending. They argue that public health spending ensures effective health services provided by the public sector, promoting the consumption of effective health services and thus improving health status [8]. Changes in the composition of public expenditure are thus emphasised. The composition of health expenditures refers to the way in which funds are distributed such as administrative costs, human resources, material endowments, public health programmes (immunization campaign for example). Following our results, it was found that endowment materials are the most effective.

Gupta et al. (2003) in studying the case of developing countries have argued that public expenditure is more effective among the poor than the non-poor. However, public spending alone does not improve health status. Governments must also ensure that health interventions reach their intended beneficiaries [31]. Therefore, quality of governance is considered not only as an important determinant of health outcomes but also of efficiency of public spending on health [12]. Studying the case of sub-Saharan African countries, Makuta and O’Hare (2015) found that the impact of health spending is higher in countries with higher quality of governance and lower in countries with lower quality of governance [12]. As indicators of the quality of governance, they considered those of the World Bank’s Worldwide Governance Indicators (WGI) (corruption control, government effectiveness, etc.). Based on the WGI indices, Madagascar is among the weakest in terms of governance, whether for political stability (-0.64), corruption control (-0.93), government effectiveness (-1.00) or regulatory quality (-0.82) in 2021. However, these indicators remain very general and not detailed. Hence the interest in analysing the bottlenecks in order to identify where the problems lie and how this poor quality of governance is reflected, particularly in the health sector. In addition to the bottlenecks already identified in most African countries such as insufficient funds, complexity in transferring funds leading to delay [14]; other bottlenecks can be added, such as misappropriation and misuse of funds, lack of control and non-regularity of donations.

Moreover, funds for health are very low in Madagascar. Indeed, they represent only 5% of the government budget in 2017. Then, it is the funds for administration and human resources that are the highest (40–60% of expenditures) [32]. Thus, although maternal and child health is a priority for the Ministry of Health [30] because of the share allocated to health, there are shortcomings in the implementation of activities.

These situations affect the quality of services and reduce the effectiveness of the health programmes. Indeed, health facilities could not invest on equipment and on medicines with the attributed funds. For maternal health, in Madagascar for example, according to a study conducted by USAID in 2018, only 51% of health facilities have access to essential elements for antenatal care (nutritional supplements, tetanus vaccine, long lasting insecticide treated nets, medicines for intermittent preventive treatment). Then, no health facility had all the basic elements for obstetric and neonatal care (delivery bed, running water, suction cup, child scale, hand vacuum cleaner, lamp, etc.). For child health, only 1% of them had different types of basic vaccines with basic materials (refrigerator at the appropriate temperature, single-use syringes, vaccine carriers, and ice packs). Finally, regarding medicines, only 47% and 54% of health facilities had essential medicines for mothers and children [33] respectively.

However, the reduction of maternal and child mortality cannot depend only on attribution of specific funds. Structural determinant such as the geographical accessibility, insufficient number of caregivers and health facilities [28] and then cultural practice do not promote access to health care [34]. In rural areas, more than 35% of the population live more than 10 km away from health facilities [35] and in 2018, there was only 1 doctor for every 13,018 inhabitants, whereas the standard recommended by the WHO is 1 doctor per 10,000 inhabitants [35].

### Effects of financing

Investment in healthcare is important for both short- and long-term benefits. Therefore, allocating more funds to health and using these funds optimally would improve health and save lives [36]. Considering the funds used for vaccination, it has been observed that this activity could save 2 to 3 million lives per year [37]. Gani (2009) finds that a 10% increase in per capita health expenditures would lead to an approximate 6.6% reduction in child mortality rate [38]. Then, according to Foster et al (2012), for every dollar spent on key interventions for reproductive, maternal, newborn and child health, about US$ 20 in benefits could be generated [39]. For the case of Madagascar, according to UNICEF (2015), when funds are insufficient or do not reach the people in charge, such as doctors or health center managers, two cases may arise: either the health activities are not maintained, or the people in charge use their own funds. However, the resources of these doctors and health center managers are already limited.

### Recommendations

#### Optimal allocation of funds and resources

Following the Abudja Conference, Madagascar have to increase the public health expenditures to at least 15% of the government budget. In addition, it is necessary to ensure optimal allocation of resources, to make health services accessible to all by implementing effective health care programmes. Optimal allocation of funds means ensuring the availability of essential equipment and medecines at the basic level and increasing the quality of the services by building staff capacity of the health facilities, particularly in rural areas. It is also necessary to ensure enhanced monitoring, supervision and audits carried out at all the levels, especially the DHOs, which are the main fund managers. It is also necessary to promote a better allocation of human resources.

In addition, considering the high degree of budget centralization, more autonomy is needed for the implementation of the part of the budget at the district and primary levels. Thus, a redistribution of funds must be processed, according to the geographical distribution of population and considering its poverty.

#### Universal Health Coverage

Madagascar implemented Universal Health Coverage (UHC) in 2015. It is "the situation in which people can obtain quality health services with costs that do not cause financial difficulties for users" (MOH, 2018). However, the implementation of the National Health Solidarity Fund (NHSF) in two pilot regions was only effective in 2018. NHSF members contribute (annual contribution of MGA 9,000 or USD 2.5) to benefit from the Minimum Package of Activities offered by health facilities (vaccination, prenatal, postnatal consultation, childbirth, transport costs, growth monitoring, micronutrient intake and oral health promotion, prevention of sexually transmitted infections and AIDS).

The main objectives of the UHC are “the financial protection of users, through health insurance system (prepaid system), the effective availability of the quality health services and the reduction of the population’s exposure to the risks affecting health through promotional and preventive activities” (MOH, 2018). One of the ways to promote UHC is thus to promote local care by the use of community health workers (CHWs) which are close to the population. Their missions are to raise awareness for care, to ensure immediate management of simple cases and to refer to the health facility in case of serious illness.

The main challenge for the UHC is its large-scale implementation. Considering the low budget capacity of the government to support health facilities’ expenses, the UHC is still uncertain.

### Limitation

Our paper has some limitations. Firstly, the studied period is limited to 2000–2017 as only these data are available at regional level. Secondly, maternal and child expenditures are not distinguished. The data relate to total health expenditures. However, as argued, there are cross-cutting expenditures, such as human resource expenditures, which consider both maternal and child health as well as health in general. Thirdly, health expenditures data in this paper refer to expenditures recorded in the Ministry of Finance and Budget’s Integrated Computerized Public Finance Management System (SIG-FP) from 2010 to 2017 (recorded public and external funds). There may be parts of off-budget data not considered. Finally, as it has been argued that there are other determinants that can explain maternal and child mortality, we have only included in the model those available by region during the studied period.

## Conclusion

This paper shows that public funding, particularly through external funding distributed by the government, contributes the most to the improvement of maternal and child health. However, equipment endowments contribute more to the improvement of the health situation and would be more effective than monetary financing. Indeed, only the relationship between health expenditures and maternal mortality is significant, although maternal health programme does not receive cash funding. Donations distributed to pregnant women during prenatal consultation and delivery has a direct impact on the use of maternal services.

Nevertheless, maternal mortality is still high and reflected in a rather low level of delivery at health facilities. Therefore, to further encourage attendance at health facilities, distributing these donations must be followed by complementary measures, including sensitization, regularity of donations, improvement of the quality of the service, and free medical costs for prenatal visit and delivery [40]. Indeed, according to UNICEF (2015), material endowments present a lower risk of funds misappropriation than funding by cash [28].

Regarding child health, interventions could be more effective if the bottlenecks are addressed by reinforcing monitoring and supervision of the use of funds at all levels of the health system. Therefore, making funds more equitably accessible to the controlling units is necessary.

## Data Availability

Data came from the Health Statistical Yearbooks of the Ministry of Health (2021). Then, we use Stata, so, the do.file is available.

## List of abbreviations

DHO: District Health Offices
EPI: Expanded Programme on Immunization
GAVI: Global Alliance for Vaccination and Immunization
MCHW: Mother and Child Health Week
MOH: Ministry of Health
SDG: Sustainable Development Goals
RHO: Regional Health Offices
TFPs: Technical and Financial Partners
UNDP: United Nations Development Programme
UNICEF: United Nations International Children’s Emergency Fund
USAID: United States Agency for International Development
WHO: World Health Organization
